# Cash, currency and COVID-19

**DOI:** 10.1136/postgradmedj-2020-138006

**Published:** 2020-05-13

**Authors:** Rimesh Pal, Sanjay K Bhadada

**Affiliations:** Endocrinology, Post Graduate Institute of Medical Education and Research, Chandigarh, Chandigarh, India; Endocrinology, Post Graduate Institute of Medical Education and Research, Chandigarh, Chandigarh, India

**Keywords:** infectious diseases

COVID-19, caused by severe acute respiratory syndrome coronavirus 2 (SARS-CoV-2) has scourged the world ever since its outbreak in December 2019, affecting over three million people and claiming more than 207 000 lives over 200 countries all over the globe. Human-to-human transmission of SARS-CoV-2 occurs primarily by fomites and by respiratory droplets. This has, in turn, fanned public concerns that cash and coins could transmit the virus as well. Central banks have reported a surge in the number of queries from the media on the safety of using cash. Internet searches pertaining to both ‘cash’ and ‘virus’ or ‘COVID’ is at an all-time high.^1^ The apprehensions have been fuelled by studies that have demonstrated the remarkable stability of SARS-CoV-2 on inanimate objects and surfaces.^2^ Hence, we have summarised the possibilities of transmission of COVID-19 via currency and the feasible alternatives amid the prevailing circumstances.

1. Does viral transmission occur through cash and coins?

Hitherto available in vitro data do suggest that human-to-human transmission of COVID-19 via cash and coins seems possible. SARS-CoV-2 has been shown to be more stable on smooth surfaces, and a detectable level of infectious virus has been recovered from banknotes and stainless steel (coins) even after 2 and 4 days of inoculation, respectively. In addition, a biphasic decay of infectious SARS-CoV-2 has been found in samples recovered from smooth surfaces, thereby further prolonging the duration of stay of the virus.^3^ However, recovery of the virus from inoculated objects in in vitro often requires elution of the virus by special techniques, like soaking the object in viral transport medium for 30 min. This might not actually reflect the potential to pick up the virus from casual contact. Nevertheless, laboratory simulations have revealed that human influenza virus, rhinovirus, rotavirus, hepatitis A virus and norovirus can be transmitted through banknotes and coins.^4^ Similar simulation data with regard to SARS-CoV-2 are lacking. Hence, in the absence of hardcore evidence, making a definite statement is not possible. However, considering the widespread magnitude of the pandemic and the remarkable stability of the virus on smooth surfaces, it would be prudent to err on the side of caution and to consider it as a potential mode of human-to-human transmission and accordingly take all necessary precautions.

2. What are the reactions of international organisations and central banks in this regard?

The WHO is uncertain about the issue and has made an uncommitted statement saying, ‘There is currently no evidence to confirm or disprove that COVID-19 virus can be transmitted through coins or banknotes’. At the same time, the WHO has advised that people wash their hands regularly after touching any frequently touched surface or object, ‘including coins or banknotes’.^5^ On the other hand, the Centers for Disease Control and Prevention is completely silent. A recently issued bulletin from the Bank for International Settlements, the body owned by 62 central banks, states that the perceived risks of transmission of COVID-19 via cash and coins, valid or not, brings the potential for digital payments to the fore.^1^ The Deutsche Bundesbank has advised that the risk of transmission via banknotes is minimal, while the South African Reserve Bank has clarified that there is no evidence of transmission by cash. The People’s Bank of China took an extreme step in February 2020 of clearing thousands of banknotes in circulation, disinfecting them with ultraviolet light, locking them up for 14 days before releasing them into circulation.

3. What are the alternatives to cash-based transactions?

Digital transactions offer a good alternative. However, use of debit/credit cards is no safe bet either, considering the remarkable stability of SARS-CoV-2 on plastic. Moreover, card transactions generally require a signature/Personal Identification Number (PIN) entry at merchant-owned devices that could be a potential source of infection. Contactless transactions are the best options under prevailing circumstances. It can be ‘card-based’ or ‘non-card based’. Card-based contactless transactions do require a credit or debit card; however, the card only needs to be tapped near a point-of-sale terminal that is equipped with contactless payment technology (also known as radio-frequency identification or near-field communication technology) and does not require the manual entry of a signature/PIN. Alternatively, one can circumvent the need to carry a card by securely storing their debit/credit card information in their smartphone or smartwatch using easily downloadable payment applications (like Apple Pay); instead of using the actual card, successful transactions can be made by tapping the smart device. Transaction sizes by this ‘tap-and-go’ method, as it is commonly called, are limited, and the allowable amount varies by country and by the bank. Recently, however, banks and card networks in the UK, Austria, Hungary, Germany, Ireland, Netherlands and elsewhere have set higher transaction limits for tap-and-go payments.^1^ Examples of non-card-based contactless transactions include digital wallets and smartphone-based payment interfaces (like Google Pay, Amazon Pay, Android Pay and Due). They can be used for making payment at shops (that often require scanning of QR codes from a distance), transferring money to near and dear ones and doing recharges, all with just a smartphone in hand. Online banking, also known as net banking, is another option that does not require handling of any cash or card and allows transaction of large amounts at a time.

4. What are the precautions and practices that can be followed while handling cash and coins?

Although the need of the hour, prioritisation of cashless–contactless transactions cannot happen overnight, specifically in developing countries, like India, where approximately 95% of all transactions are still made using cash. The more the number of transactions, the longer the banknotes and coins remain in circulation, and the more opportunity for them to become contaminated. Hence, proper precautions should be taken while dealing with banknotes/coins. Hands should be promptly washed with soap and water soon after handling cash/coins. The use of gloves and alcohol-based sanitisers (70% alcohol) is advisable for people who have to frequently handle cash, for instance, shopkeepers, but even then, it is advised that they do not touch their face and dispose of gloves safely after use. Cash/coins can be left untouched for a week before reuse. In addition, coins can be easily washed with soap and water. Banknotes can be ironed (preferably steam press) with temperature set between the ‘silk’ and ‘rayon’ setting (~148°C to 190**°**C). Such high temperatures would be expected to kill the SARS-CoV-2 as the virus gets readily inactivated at a temperature of 70**°**C.^3^ Lastly, installments dealing with large transactions, like central banks, can consider disinfecting cash and coins using ultraviolet light.

In conclusion, banknotes and coins should be considered as potential sources of transmission of the novel SARS-CoV-2. Further laboratory stimulation data might help resolve the issues. Until then, handling of cash and coins should be done with utmost precaution; cashless and contactless transactions using online banking and digital wallets should be pursued wherever possible.

## References

[1] Auer R, Cornelli G, Frost J. Covid-19, cash, and the future of payments 2020.

[2] van Doremalen N, Bushmaker T, Morris DH, et al. Aerosol and surface stability of SARS-CoV-2 as compared with SARS-CoV-1. N Engl J Med 2020;382:1564–7. 10.1056/NEJMc2004973 32182409 PMC7121658

[3] Chin AWH, Chu JTS, Perera MRA, et al. Stability of SARS-CoV-2 in different environmental conditions. Lancet Microbe 2020. 10.1016/S2666-5247(20)30003-3 PMC7214863 32835322

[4] Angelakis E, Azhar EI, Bibi F, et al. Paper money and coins as potential vectors of transmissible disease. Future Microbiol 2014;9:249–61. 10.2217/fmb.13.161 24571076

[5] WHO . Q&A on Coronaviruses (COVID-19). Available: https://www.who.int/news-room/q-a-detail/q-a-coronaviruses [Accessed 20 Apr 2020].

